# Physical Activity Monitoring in Adolescents and Young Adults With Cancer: Observational Feasibility Study

**DOI:** 10.2196/87591

**Published:** 2026-04-13

**Authors:** Savannah V Rauschendorfer, Beatrice Cantoni, Michelle Audirac, Susan C Gilchrist, J Andrew Livingston, Michael E Roth, Corwin M Zigler, Eugenie S Kleinerman

**Affiliations:** 1 Health, Human Performance, and Recreation, Baylor University, 1311 S 5th Street, Waco, TX, 76706, United States, 1 3175045175; 2 Department of Statistics and Data Sciences, The University of Texas at Austin, Austin, TX, United States; 3 Department of Biostatistics, Harvard University, Boston, MA, United States; 4 Department of Medicine, Division of Cardiology, University of North Carolina School of Medicine, Chapel Hill, NC, United States; 5 Department of Sarcoma Medical Oncology, The University of Texas MD Anderson Cancer Center, Houston, TX, United States; 6 Department of Pediatrics, The University of Texas MD Anderson Cancer Center, Houston, TX, United States; 7 Department of Biostatistics, Brown University, Providence, RI, United States

**Keywords:** sarcoma, adolescents and young adults, physical activity tracking, wearable device, chemotherapy

## Abstract

**Background:**

There is a growing interest in characterizing the relationship between long-term physical activity (PA) habits and cancer-related outcomes such as treatment-related toxicities, recurrence, and complications. Wearable devices can provide critical information to achieve this goal; however, inferences are significantly influenced by device wear adherence.

**Objective:**

This study aimed to assess the feasibility of using wearable devices to monitor short- and long-term PA in adolescent and young adult (AYA) patients with sarcoma during and after chemotherapy in a free-living environment and evaluate the ability to accurately capture changes in PA over 3 years.

**Methods:**

A total of 63 AYA patients with sarcoma were provided with a Fitbit Charge 3 to track daily steps, sedentary time, and heart rate for up to 3 years.

**Results:**

On average, during the first 30 days of follow-up, 57.1% (36/63) of patients wore their device at least 10 hours per day, and only 23.8% (15/63) of patients wore their devices thereafter. Patients spent a mean of 80% (SD 11%) of their day in a sedentary state. Despite low adherence, daily step count trends increased over time.

**Conclusions:**

This study highlights the adherence challenges met with longitudinal PA monitoring in AYA patients with sarcoma. Wearer discomfort, lost devices, and lack of data uploading compliance contributed to data missingness and attrition. Caution is warranted when relying on wearable activity trackers to inform program decisions, accurately assess PA outcomes, and monitor program adherence longitudinally without consideration of wearer bias. Alternative methods that would be more broadly accepted by AYA patients for effective long-term monitoring should be considered.

## Introduction

Adolescent and young adult (AYA) patients who receive high doses of anthracycline treatment such as doxorubicin are at high risk of long-term cardiac morbidity and mortality [1]. Physical activity (PA) has been previously demonstrated to protect against and mitigate doxorubicin-induced cardiotoxicity [2-4]. As a result, exercise is often proposed as a nonpharmacological early prevention intervention to combat cardiovascular side effects of cancer therapy. There is a growing interest in characterizing the relationship between long-term PA habits and cancer-related outcomes such as treatment-related toxicities, recurrence, and complications. Furthermore, there is interest in understanding how PA interventions may modify these crucial outcomes. Wearable devices are widely used for quantification of PA [5]. Among the demographic of AYA patients with sarcoma who receive high-dose anthracyclines, little is known regarding the feasibility of using wearable devices to monitor PA longitudinally for associations with late cancer-related outcomes.

In addition to wearable devices being capable of providing long-term activity data [6-8], it has been well established that PA monitoring can inform exercise prescription decisions and quantify exercise intervention participation [9-11]. Information gathered from PA monitoring devices aids in designing tailored interventions for specific patient populations of interest. Exercise intervention programming and resultant prevention success hinge on the current behaviors, interest, and activity levels of a given cohort of individuals [12]. On the basis of the target population, exercise prescription in terms of frequency, intensity, time, and type can be highly variable. Among AYA patients with cancer, there is a lack of consensus on frequency, intensity, time, and type parameters to be used in patients receiving therapy [13]. Information gathered from regular activity levels may indicate optimal timing as well as intensity for a practical PA intervention. Therefore, recording PA in AYA patients undergoing chemotherapy is essential for the design of a feasible exercise regimen to improve adherence probability in a randomized trial.

The lack of participant willingness to wear and interact with PA tracking devices longitudinally can present a significant challenge for creating exercise interventions, monitoring program adherence, conducting unbiased data interpretation, and deducing critical scientific conclusions for determining the impact of exercise interventions on cancer-related toxicities. Researchers have called for investigations that evaluate the long-term adherence to and sustainability of activity-monitoring interventions using wearables [14-16]. Therefore, the purpose of this study was to evaluate the feasibility of assessing short- and long-term PA during and after chemotherapy using a wearable device in AYA patients with sarcoma in a free-living environment and determine whether such data can accurately describe the evolution of PA levels of AYA patients over the span of 3 years.

## Methods

### Patient Recruitment

PA was assessed as a component of a primary cardiotoxicity investigation. Patients between the ages of 15 and 39 years at the time of study entry were recruited at MD Anderson Cancer Center. The study follow-up period for the primary cardiotoxicity end points was 2 years after recruitment. Cardiotoxicity end points and sample size calculations were based on evidence that demonstrated significant cardiac remodeling 2 years after diagnosis [17]. However, patients were informed that the Fitbit study monitoring period would last up to 3 years from enrollment, wherein data would be collected passively without intervention to maximize the information gained from the wearable device and gaining new information from it.

Inclusion criteria were patients with a histologically confirmed diagnosis of sarcoma (including either soft tissue or bone sarcoma) and anticipated total doxorubicin dose of 300 mg/m^2^ or higher or other anthracycline with doxorubicin equivalence of 300 mg/m^2^ or higher as determined by the treating physician. Moreover, individuals included in the study must indicate that they were willing to wear and upload data from the PA wearable device throughout the study period. Patients were excluded if there was any condition that compromised the ability of the patient or their legally recognized representative to give written informed consent and/or comply with the study. Patients with a history of cardiac morbidity were also excluded from study participation.

### Ethical Considerations

Informed consent was obtained from all participants included in the study. The study was approved by the MD Anderson Cancer Center institutional review board (PA18-0462). All study procedures were conducted in accordance with the ethical standards of the Declaration of Helsinki. The privacy and confidentiality of the research participants’ data and identity were maintained. Patients were allowed to keep the Fitbit Charge 3 (Google) device after study conclusion or discontinuation. No other patient incentive was provided for participation.

### PA Tracking

PA was assessed using an electronic wearable activity-monitoring device (Fitbit Charge 3 provided by the Department of Pediatrics of MD Anderson Cancer Center). Fitbit devices are widely used and well known for their commercial popularity, high wear compliance, and strong correlation with research-grade monitoring devices [15,18]. The Fitbit Charge 3 was selected based on the resources available for device purchase and implementation [19], as well as for its demonstrated significant correlation with research-grade PA monitoring devices such as the ActiGraph wGT3X-BT [18]. After consent, patients were given a Fitbit device and went through an educational introduction to the device whereby a study coordinator explained how to wear it and charge it and aided in downloading the Fitbit app to the participants’ mobile devices or tablets for device data synchronization. Patients were asked to wear the device for a minimum of 6 months and were instructed to wear it at all times unless they were charging it. Participants were additionally informed that the study monitoring period would last a maximum of 3 years. No PA-specific instructions were given in order to to assess changes in PA without an intervention during and after chemotherapy. The Fitbit device was used to track aerobic PA, which included minute-level measures of steps; heart rate (HR); and a classification of activity as sedentary, light, moderate, or active. The device was linked to Fitabase (Small Steps Labs LLC), a database that stores activity data on a secure online website. Data storage on the Fitabase platform was participant dependent via app synchronization. Therefore, data were reviewed routinely throughout the study period. Study coordinators attempted to contact participants who did not upload data for 10 of the previous 14 days for participant retention. There were no device-integrated automatic or proactive reminders to participants to wear or synchronize their devices.

### Device Wear Time Parameters

Whether the device was being worn in at a given minute was determined based on (1) whether the patient’s device logged any data during the given day and (2) applying the algorithm developed by Choi et al [20] to consider the device worn during minutes for which there was an HR measurement or during minutes of spurious missing HR. PA was summarized between the hours of 8 AM and 8 PM, with daily summaries calculated only for “valid days,” defined as days during which the device was worn for at least 10 hours between 8 AM and 8 PM per best practice consensus for waking day sampling [19]. The 8 AM to 8 PM time window has been previously used and defined as a standard waking or daytime period used to monitor PA [21-23].

### Statistical Analysis

Study device wear patterns were summarized by calculating the proportion of participants enrolled on a given follow-up day who wore the Fitbit device for at least 10 hours between 8 AM and 8 PM (a “valid day”). Daily PA summaries for each patient were calculated as the total number of steps, average daily HR, and proportion of time classified as sedentary activity among valid days of device wear. Means are reported with the SD, and medians are reported with the IQR. To evaluate changes in PA over time, a mixed-effects linear regression model with normally distributed random intercepts and slopes was fit to model trends in daily step count. This model specified patient-specific time trends, various patient characteristics (sex, tumor location, BMI, and age), and treatment status (during or after chemotherapy treatment) and adjusted for day of the week variability. The follow-up day was calculated as the day from primary cardiotoxicity study enrollment.

## Results

### Overview

A total of 75 patients were eligible, recruited, consented for participation, and enrolled in the study. In total, 84% (63/75) of these patients (33/63, 52.4% male and 30/63, 47.6% female) had valid Fitbit data that were included in the analysis (Table 1). The reasons for 16% (12/75) of the individuals not logging Fitbit data were variable; however, most related to inability or unwillingness to use the Fitbit device and/or its supporting app or study withdrawal before setting up the Fitbit device. The mean age and BMI of the cohort were 24 (SD 7.4) years and 27.7 (SD 10.0) kg/m^2^, respectively. The most common types of sarcoma diagnosis were osteosarcoma (18/63, 28.6%) and synovial sarcoma (13/63, 20.6%), and the most common locations of the primary tumor were the trunk (33/62, 53.2%) and lower limb (21/62, 33.9%; Table 1). Patients were enrolled in the primary study for a median of 730 (IQR 622-730) days. The median number of days that a patient logged Fitbit data was 205 (IQR 58-352).

Over the study duration, 11.1% (7/63) of the wearable devices were lost, and 3.2% (2/63) were broken. All were replaced by the study team. Among the 63 patients, there were 5 (7.9%) reports of wearer discomfort due to skin sensitivity wherein patients subsequently stopped wearing the activity tracker. In several instances, wearers forgot to synchronize the device for data transfer, making consistent data collection a recurrent challenge for the study team.

**Table 1.** Patient characteristics (n=63).

	Values
Duration of study enrollment (days), mean (SD)	630 (208)
Duration of study enrollment (days), median (IQR; range)	730 (622-730; 3-1006)
Duration of Fitbit participation (days), mean (SD)	245 (226)
Duration of Fitbit participation (days), median (IQR; range)	205 (58-352; 2-950)
Age (years), mean (SD)	24 (7.4)
Age (years), median (IQR; range)	23 (18-31; 15-39)
BMI (kg/m^2^), mean (SD)	27.7 (10.0)
BMI (kg/m^2^), median (IQR; range)	24.3 (21.0-31.0; 15.4-65.7)
Total doxorubicin dose (mg/m^2^), mean (SD)	354.27 (125.84)
Total doxorubicin dose (mg/m^2^), median (IQR; range)	379.80 (299.25-446.50; 25.00-633.37)
Radiation, n (%)	
Yes	26 (41.3)
No	37 (58.7)
Surgery, n (%)	
Yes	42 (66.7)
No	21 (33.3)
Sex, n (%)	
Female	30 (47.6)
Male	33 (52.4)
Sarcoma diagnosis, n (%)	
Angiosarcoma	2 (3.2)
Ewing sarcoma	9 (14.3)
Liposarcoma	5 (7.9)
Osteosarcoma	18 (28.6)
Rhabdomyosarcoma	4 (6.3)
Soft tissue sarcoma	3 (4.8)
Synovial sarcoma	13 (20.6)
Other	9 (14.3)
Tumor location (n=62), n (%)	
Trunk	33 (53.2)
Lower limb	21 (33.9)
Upper limb	3 (4.8)
Other	5 (8.1)

### Device Wear Summary

**Figure 1.**
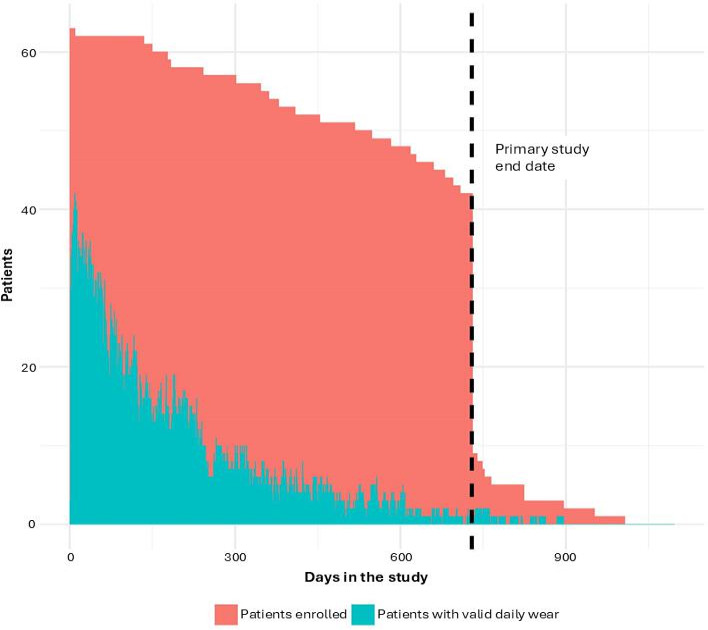
Number of patients enrolled (red) and number of patients exhibiting at least 10 hours of Fitbit wear time between 8 AM and 8 PM (“valid day”; blue) across their days in the study. Given that physical activity was assessed as a component of a primary cardiotoxicity investigation, the primary cardiotoxicity study end point has been indicated in the figure (black dotted line). Patients were informed that the Fitbit study monitoring period would last up to 3 years from enrollment, wherein data would be collected passively without intervention.

**Figure 2.**
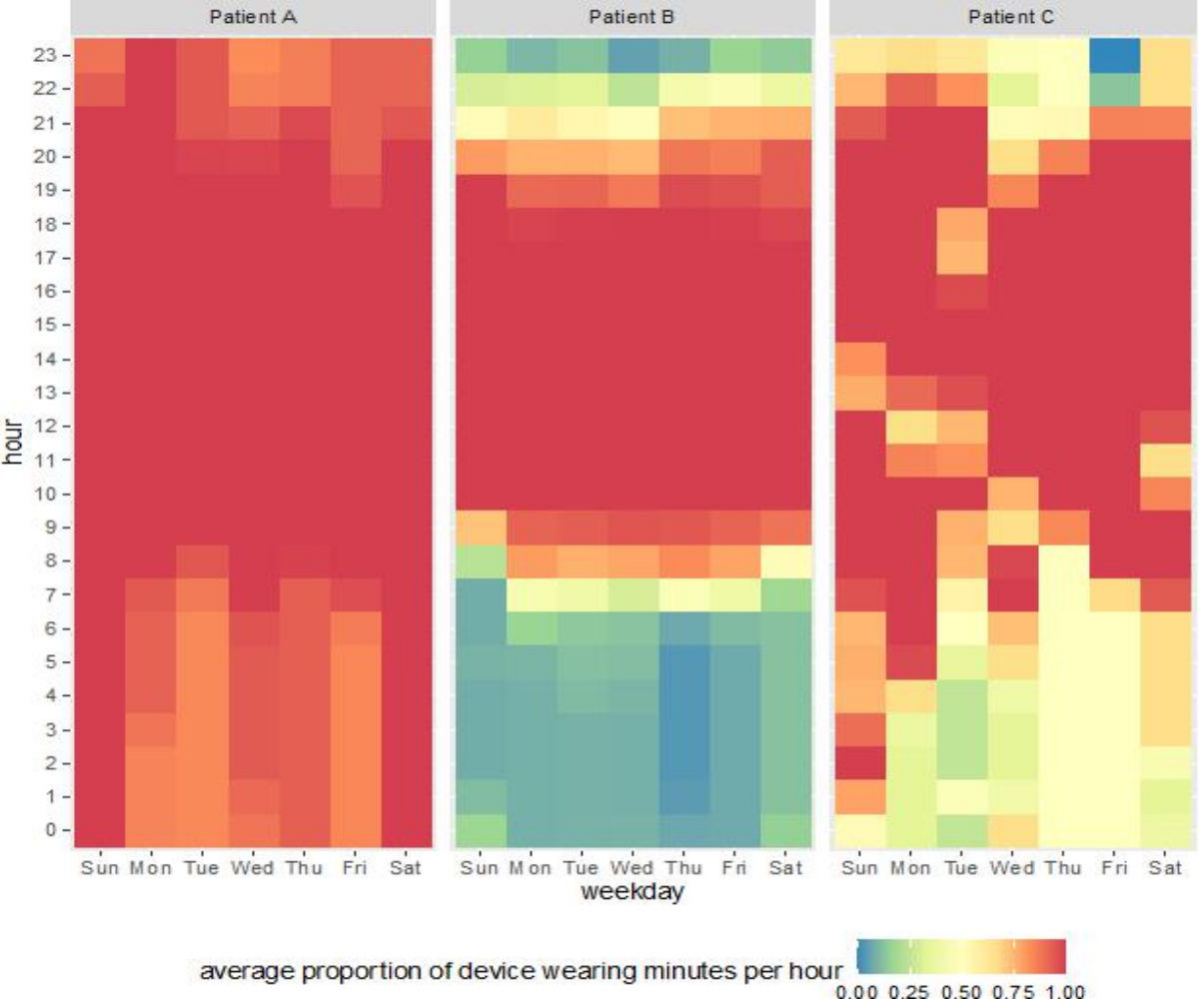
Proportion of device wear time during specific hours of days of the week across 3 anonymized patients.

The highest proportion of device wear occurred on day 9 of follow-up, when 66.7% (42/63) of study participants exhibited valid wear of at least 10 hours between 8 AM and 8 PM. All other study days saw fewer patients with valid device wear, which decreased as the study progressed. Allowing for a 7-day lag to set up the Fitbit app, the average proportion of patients exhibiting valid daily wear was 57% during follow-up days 8 to 30, with the proportion of valid wear averaging 24% during days 31 to 365 and 6% from day 366 to the primary cardiotoxicity study end point of 2 years. Device wear during the study period is summarized in Figure 1. Moreover, patients demonstrated intervariability for daily patterns of device wear. Figure 2 shows key examples of the heterogeneity of wear patterns across different hours of the day and days of the week for 3 anonymized patients.

### PA During Device Wear

**Figure 3.**
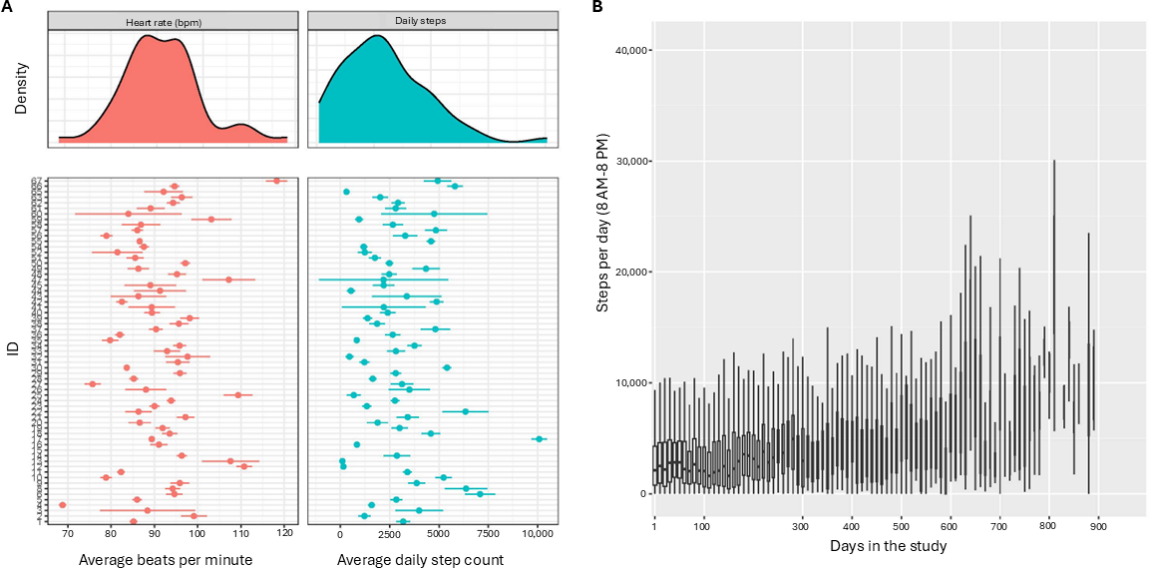
(A) Summary of mean daily steps and heart rate: density of average daily heart rate (top left) and step count (top right) across all study participants and individual estimates and 95% CIs for mean daily heart rate (lower left) and daily step count (lower right) for individual study participants. (B) Daily steps by length of time in the study: box plots of steps per day for all participants exhibiting valid daily wear for each study follow-up day. The width of the box plots is proportional to the number of study patients logging Fitbit data on that follow-up day. bpm: beats per minute.

Table 2 summarizes the overall daily HR, step count, and proportion of time between 8 AM and 8 PM spent in the sedentary activity category among available valid days of device wear. The cohort was found to have a mean daily HR of 91 (SD 8.6) beats per minute and a mean daily step count of 2970 (SD 1902) steps. Moreover, patients spent a mean of 80% (SD 11%) of their day in a sedentary activity state. Figure 3A summarizes observed heterogeneity in PA across patients in terms of daily steps and HR during valid days for the entire study sample and for each patient individually. Table 3 describes Fitbit PA measures during patients’ chemotherapy treatment period and after completion of chemotherapy. On average, and as expected, patients exhibited slightly less steps during chemotherapy (mean 2714, SD 1747 vs 3676, SD 2743 after), with comparable average HR and proportion of time spent sedentary. Importantly, we additionally found that 98.4% (62/63) of the patients had valid Fitbit wear during chemotherapy, whereas postchemotherapy summaries are based on only 68.3% (43/63) of the patients, who had valid Fitbit wear after completion of chemotherapy.

Figure 3B depicts the distribution of average daily step count across all participants exhibiting valid daily wear on each day of study follow-up, showing that the average daily step count among device wearers tended to increase over the course of the study. To further evaluate changes in daily step count over time, a mixed-effects regression model was fit with patient-specific random intercepts and slopes for follow-up time and adjustment for day of the week, patient sex, BMI, and tumor location, as well as whether the steps were recorded during or after chemotherapy treatment. Regression coefficient estimates from this model are reported in Table 4. This model indicated that, on average, across all patients, there was no significant trend in daily step counts as patients remained in the study for longer (*P*=.85), although there was substantial heterogeneity in patient-specific daily step trends—evidenced by a high variance of the patient-level random intercepts. The model also estimated that daily step count was 487 steps lower on average during chemotherapy treatment than after completion of chemotherapy (*P*<.001). With the exception of patient age, there was no evidence that any other patient characteristic or variable was significantly associated with daily step counts after accounting for chemotherapy, day of the week, and patient-specific time trends.

**Table 2.** Fitbit physical activity summary (n=63).

	Mean (SD; range)
Daily HR^a^ (bpm^b^)	91 (8.6; 69-118)
Daily steps	2970 (1902; 100-10,082)
Daily proportion of sedentary activity (%)	80 (0.11; 37-98)

a HR: heart rate.

b bpm: beats per minute.

**Table 3.** Fitbit physical activity summary during and after chemotherapy.

	During chemotherapy (n=63)	After chemotherapy (n=43)
	Mean (SD; range)	Mean (SD; range)
Daily HR^a^ (bpm^b^)	91 (8.1; 70-114)	92 (11; 69-118)
Daily steps	2714 (1747; 82-9909)	3676 (2743; 105-12,533)
Daily proportion of sedentary activity	0.81 (0.11; 0.27-0.98)	0.79 (0.11; 0.56-0.98)

a HR: heart rate.

b bpm: beats per minute.

**Table 4.** Estimated regression coefficients from the linear mixed-effects model of daily steps during the study period^a^.

Variable	Estimate (SE)	*P* value
Intercept	636.628 (1535.538)	.68
Days in the study	−0.345 (563.422)	.85
During chemotherapy	−487.326 (115.124)	<.001
Sex: male	625.104 (498.854)	.22
Age	77.911 (34.891)	.03
BMI	15.181 (25.771)	.56
Tumor location: lower limb	−184.588 (923.439)	.84
Tumor location: trunk	−286.307 (895.38)	.75
Tumor location: upper limb	1549.331 (1363.375)	.26

a Estimates represent the average difference in daily steps associated with a 1-unit increase in each individual feature controlling for all other variables presented in the model table, patient-specific trends in daily steps, and day of the week. The sex coefficient is the difference between male and female patients. Tumor location estimates are compared to “other” locations.

## Discussion

### Principal Findings

Given the popular desire by investigators to quantify longitudinal PA levels and changes in PA among AYA patients with cancer for the evaluation of the relationship between PA and cancer-related clinical outcomes, we investigated the practicality of assessing longitudinal PA using a wearable device in AYA patients with sarcoma who received high-dose doxorubicin in a free-living environment.

We found that, although participants were enrolled in the study for an average period of 630 (SD 208; median 730, IQR 622-730) days, PA wearable device data were logged for less than half of that time (mean 245, SD 226; median 205, IQR 58-352 days). Devices were worn and tolerated by an average of 57% of AYA patients for PA monitoring and data logging for at least 10 hours from 8 AM to 8 PM up to day 30 of study enrollment. Device wear declined drastically to 24% of patients on average after follow-up day 30. On the basis of AYA patient device wear patterns from this study, we identified that 30 days may be the upper limit for study duration in investigations that aim to use wearable devices such as Fitbit in similar cohorts, particularly without more active efforts to encourage device wear. We do not believe that these findings are exclusive to Fitbit device monitoring. Importantly, there is significant heterogeneity in the devices used in the field of PA monitoring [15]. We expect that our findings in AYA patients with sarcoma likely extend to the vast majority of devices. This study did not provide proactive reminders to participants to wear or synchronize the devices. Due to this passive monitoring modality, patients lacked routine motivational encouragement to wear their devices. Previous studies have demonstrated a significant positive impact of using SMS text message–, app-, and device-based reminders, notifications, and encouragement on PA-related behaviors, self-management, and adherence [24-26]. Therefore, motivational tactics should be considered for future investigations to modify behavioral patterns that augment device wear.

The practicality of using wearable devices in AYA patients with sarcoma appears to be greatly challenged by attrition and wearer difficulties. During the study period, qualitative reports of wearer and user challenges such as wearer discomfort, lost devices, and lack of data uploading compliance were identified as primary contributors to data missingness and participant attrition. Considering the high use of technology and mobile devices among AYA individuals [27,28], future studies that aim to monitor PA for longer periods should consider individual PA monitoring preferences and the use of digital health and mobile app alternatives rather than wearable devices to leverage current technology routinely used by this population [28]. Moreover, future studies should consider individual and treatment-related barriers such as side effects (eg, drug-induced skin sensitivity), psychological motivation, time availability, and technological challenges and preferences that AYA patients may face during monitoring periods that may largely impact adherence [14-16].

Our secondary longitudinal outcome evaluated what happened to the PA levels of AYA patients with sarcoma during and after exposure to high-dose chemotherapy. We found that, on average, across the subset of patients who persistently wore their devices both during and after chemotherapy, AYA patients performed 487 fewer steps during chemotherapy compared to after chemotherapy. These findings of reduced PA during chemotherapy are in agreement with those of several previous studies in the field [13,29,30]. After chemotherapy, the activity levels were observed to increase longitudinally over time (measured via step counts). However, such an inference is limited to the subset of patients who persistently wore their devices during and after chemotherapy. This key finding highlights the potential risk for self-selection or wearer bias and subsequent incorrect study conclusions. Individuals who are more physically active may be more likely to wear an activity-tracking device, which may result in inflation or overestimation of average PA. Future investigators should be wary of this inference limitation when collecting PA data using wearable devices.

Overall, individuals within the cohort demonstrated variable patterns of wear (length of time and days of the week) and heterogeneity in activity level. On average, the patients spent 80% (SD 11%) of their day sedentary. Interestingly, this cohort of AYA patients with sarcoma was found to have substantially lower PA levels (steps) than comparative reports. Previous studies have found that AYA patients with bone, soft tissue, and additional mixed cancer diagnoses undergoing variable therapies and procedures recorded an average of 5699 to 6434 steps per day and a notable range of patient variability [13]. In contrast, this cohort spent 80% (SD 11%) of their activity time between 8 AM and 8 PM sedentary and recorded an average of 2714 (SD 1747) steps during chemotherapy and 3676 (SD 2743) steps after chemotherapy completion. This difference in PA level may be explained by lifestyle behaviors, cancer location, surgery, radiation, and/or high anthracycline chemotherapy dosages. In total, 33.9% (21/62) of the patient population in our study had lower-extremity tumors, which may cause reduced lower body function and PA. Additionally, anthracycline chemotherapy has been observed to cause significant skeletal muscle weakness and fatigue [31]. Similar deleterious effects on skeletal muscle function and reduced PA are commonly reported after both radiation therapy and significantly reported after surgery.

Several limitations of this study should be considered, such as device selection, confounding factors, patient education and incentives, and potential biases. This study used the Fitbit Charge 3 device to quantify PA. While the Fitbit Charge 3 is known to be significantly correlated with research-grade devices such as the ActiGraph wGT3X-BT, it has been previously demonstrated that Fitbit may comparably overestimate PA [18]. The sedentary PA level among the cohort may be in part due to a combination of cancer location and fatigue. This study was not designed to determine the *impact* of either of these potentially confounding factors but, rather, report on PA tracker wear patterns and the resultant activity data and assess the usefulness of a PA monitoring device for future studies investigating the impact of PA on cancer-related clinical outcomes such as cardiac morbidity and fatigue. Moreover, this study was designed as an observational feasibility study. Our study was not designed with the intention to parse or investigate the cancer-related psychosocial or behavioral motivations that may impact the use of wearables. The psychological impact of cancer diagnoses and care should be considered in future wearable device research. Although device familiarization and patient education were provided for the use of wearable devices in this study, future studies should consider continuing education as a necessary component to ensure consistent use and study validity. This study did not offer patient incentives beyond allowing participants to keep the wearable device. Monetary or other incentives should be considered to increase adherence past 30 days and encourage PA throughout the study [32]. When interpreting patterns from wearable devices, it is essential to consider the possibility that device wear is related to PA, meaning that individuals who consistently adhere to wearing devices may differ systematically in their activity levels, potentially skewing data toward either higher or lower levels of PA. We restricted PA measurement to “valid days” of greater than or equal to 10 hours of wear during waking hours (8 AM-8 PM) to ensure that PA summaries actually represented daily behavior. Nonetheless, patients wore their devices for variable lengths of time throughout the study follow-up, implying that cross-sectional summaries at different study time points (eg, Table 3 and Figure 3B) cannot distinguish between behavior *changes* among persistent device wearers and behavioral *differences* between persistent wearers and patients who stopped wearing their devices. The mixed-effects regression model used in this study was designed to measure individual patient behavior changes over the time when the device was worn. The lack of significant trend in daily steps from this model suggests that individual patients did not tend to change their behavior over time. The apparent increase in daily steps among all patients with valid wear on a given follow-up day depicted in Figure 3B suggests that patients who wore their devices longer into the study were those who were more physically active.

### Conclusions

This study demonstrated the need to identify alternative methods to a wearable device that would be more acceptable to AYA patients with cancer for monitoring PA longitudinally. On the basis of AYA patient device wear patterns from this study, we identified 30 days as a potential upper limit for study duration when using wearable devices such as Fitbit to accurately represent free-living environment PA without increased risk of biases. This study additionally confirmed poor PA levels among AYA patients with sarcoma. Importantly, future studies should consider AYA patient preferences for PA quantification and reporting, as well as study designs that implement continued patient education, regular participant notifications and reminders, and adherence incentives to ensure optimal longitudinal data collection and study success.

## Abbreviations

AYA: adolescent and young adult
HR: heart rate
PA: physical activity
